# Author Correction: The role of mPFC and MTL neurons in human choice under goal-conflict

**DOI:** 10.1038/s41467-020-17982-z

**Published:** 2020-08-10

**Authors:** Tomer Gazit, Tal Gonen, Guy Gurevitch, Noa Cohen, Ido Strauss, Yoav Zeevi, Hagar Yamin, Firas Fahoum, Talma Hendler, Itzhak Fried

**Affiliations:** 1 Sagol Brain Institute Tel Aviv, Wohl Institute for Advanced Imaging, Tel Aviv Sourasky Medical Center, Tel Aviv, Israel; 2 Sackler Faculty of Medicine, Tel Aviv University, Tel Aviv, Israel; 3 Department of Neurosurgery, Tel Aviv Sourasky Medical Center, Tel Aviv, Israel; 4 School of Psychological Sciences, Faculty of Social Sciences, Tel Aviv University, Tel Aviv, Israel; 5 Functional Neurosurgery Unit, Tel Aviv Sourasky Medical Center, Tel Aviv, Israel; 6 Sagol School of Neuroscience, Tel Aviv University, Tel Aviv, Israel; 7 Department of Statistics and Operation Research, Tel Aviv University, Tel Aviv, Israel; 8 Epilepsy Unit, Department of Neurology, Tel Aviv Sourasky Medical Center, Tel Aviv, Israel; 9 Department of Neurosurgery, David Geffen School of Medicine, University of California Los Angeles, Los Angeles, CA USA

**Keywords:** Motivation, Reward

Correction to: *Nature Communications*10.1038/s41467-020-16908-z, published online 24 June 2020.

The original version of this Article contained an error in the Acknowledgements section. This section was missing the sentence ‘Special thanks to Dr. Eran Eldar (Hebrew University of Jerusalem) for the inspiration and access to early versions of the PRIMO game.’ The error has now been corrected in both the PDF and HTML versions of the Article.

