# Supplementary material for: The role of mPFC and MTL neurons in human choice under goal-conflict
Source: Nat Commun. 2020 Jun 24;11:3192. doi: 10.1038/s41467-020-16908-z (PMC7314808; doi:10.1038/s41467-020-16908-z)
Supplement: Supplementary file 1 — Supplementary Information [file 41467_2020_16908_MOESM1_ESM.pdf]

Supplementary Information

The Role of mPFC and MTL Neurons in Choice under Goal Conflict in Humans

Gazit et al.

Supplementary Data

Supplementary Table 1: Electrode locations

PATIENT	MNI X	MNI Y	MNI Z	AREA
**1**	20	2	-22	'RA'
**1**	22	-14	-26	'RH'
**1**	24	-18	-14	'RH'
**1**	4	38	0	'RCC'
**1**	8	44	22	'RdmPFC'
**1**	0	0	40	'RCC'
**2**	-22	-4	-22	'LA'
**2**	-30	-22	-10	'LH'
**2**	-5	36	2	'LCC'
**2**	18	-4	-22	'RA'
**2**	28	-16	-14	'RH'
**2**	2	38	0	'RCC'
**2**	4	20	38	'RdmPFC'
**3**	-2	48	6	'LCC'
**3**	-24	-10	-24	'LH'
**3**	-10	32	22	'LCC'
**3**	4	46	2	'RdmPFC'
**3**	4	24	36	'RdmPFC'
**3**	14	-4	-20	'RA'
**3**	16	-16	-16	'RH'
**4A**	28	-24	-16	'RH'
**4A**	18	-4	-24	'RA'
**4A**	2	36	0	'RCC'
**4A**	6	34	40	'RdmPFC'
**4A**	10	44	20	'RdmPFC'
**4A**	-20	-26	-20	'LH'
**4A**	-8	44	2	'LCC'
**4A**	-8	32	32	'LdmPFC'
**5**	-22	-6	-22	'LA'
**5**	-26	-24	-14	'LH'
**4B**	20	-6	-22	'RA'
**4B**	2	44	36	'RdmPFC'
**4B**	6	42	12	'RCC'
**4B**	6	16	36	'RCC'
**4B**	4	60	14	'RdmPFC'
**6**	18	-24	-20	'RH'
**6**	26	-14	-18	'RH'
**6**	18	-2	-20	'RA'
**6**	-24	-22	-20	'LH'
**6**	-26	-12	-18	'LH'
**6**	-22	-4	-36	'LA'
**6**	-10	34	-4	'LCC'
**6**	-6	34	16	'LCC'
**7**	-10	42	-6	'LCC'
**7**	-6	24	42	'LdmPFC'
**7**	2	30	-8	'RCC'
**7**	2	36	36	'RdmPFC'
**8**	-6	42	-4	'LCC'

**8**	24	-28	-8	'RH'
**8**	6	42	-2	'RCC'
**8**	-16	0	-16	'LA'
**8**	-8	32	16	'LCC'
**9**	28	-24	-10	'RH'
**9**	24	0	-22	'RA'
**9**	-18	-2	-20	'LA'
**9**	2	26	20	'RCC'
**10**	2	36	0	'RCC'
**10**	4	50	38	'RdmPFC'
**10**	-2	38	8	'LCC'
**10**	-4	52	32	'LdmPFC'
**10**	-4	16	28	'LCC'
**10**	-4	16	54	'LdmPFC'
**11**	-4	50	26	'LdmPFC'
**11**	-4	20	42	'LdmPFC'
**11**	0	46	26	'RdmPFC'
**12**	-22	-6	-20	'LA'
**12**	-26	-22	-18	'LH'
**13**	18	-3	-24	'RA'
**13**	23	-14	-20	'RH'
**14**	23	-12	-26	'RH'
**14**	8	27	24	'RCC'

RA = Right Amygdala, LA = Left Amygdala, RH = Right Hippocampus. LH - Left Hippocampus, RdmPFC = Right dorso-medial Prefrontal Cortex, LdmPFC = Left dorso-medial Prefrontal Cortex, RCC = Right Cingulate, LCC = Left Cingulate

<u>Supplementary Table 2</u>: Neural yield per area

PATIENT\AREA	AMYGDALA	HIPPOCAMPUS	DMPFC	CC
**1**	3	4	7	1
**2**	13	7	1	10
**3**	3	3	1	11
**4A**	5	2	13	7
**4B**	10	0	10	21
**5**	4	2	0	0
**6**	9	18	0	11
**7**	0	0	4	5
**8**	11	9	0	13
**9**	11	5	0	10
**10**	0	0	19	17
**11**	0	0	8	0
**12**	9	4	0	0
**13**	0	6	0	1
**14**	1	1	0	0
**TOTAL**	69	50	63	106

DMPFC – dorson-medial prefrontal cortex; CC – cingulate cortex

Supplementary Table 3: Balanced paired comparisons of difference punishment outcomes

Reward VS. Punishment without reward	Reward	Punishment without reward	Both
MTL	11	11	2
mPFC	8	21	3

Reward VS. Punishment failed approach	Reward	Punishment failed approach	Both
MTL	7	5	1
mPFC	8	14	2

Uncontrolled punishment VS. Punishment without reward	Uncontrolled punishment	Punishment without reward	Both
MTL	8	13	0
mPFC	6	21	1

Uncontrolled punishment VS. Punishment failed approach	Uncontrolled punishment	Punishment failed approach	Both
MTL	2	6	0
mPFC	6	18	0

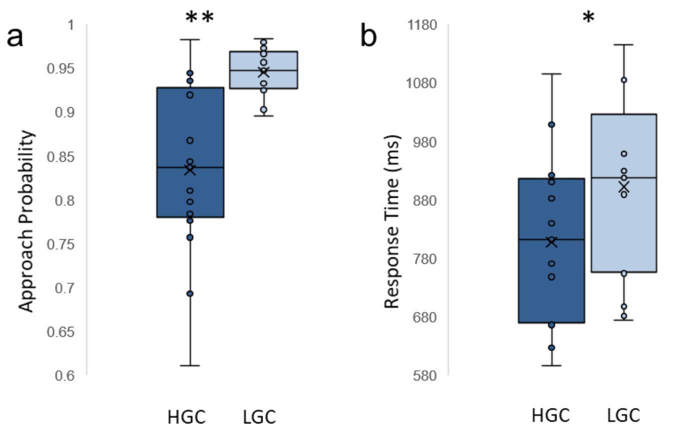

<u>Supplementary Figure 1</u>: Behavioral results: (a) Boxplots of approach probability (i.e.avatar approached the coin) for the High (dark blue) and Low (light blue) Goal Conflict (HGC, LGC) conditions in the game. Two-sided paired t-test of N=15 implantations, p=0.0003, mean different =0.11, CI = (0.06,0.16) (b) Boxplots of time between reward cue appearance and the first avatar movement towards the goal, for the high (dark blue) and low (light blue) GC conditions. Two-sided paired t-test of N=15 implantations, p=0.003, mean difference=95, CI = (37.2,152.9). Asterisk and double Asterisk denotes significant two-sided t-test at p<0.01 and p<0.001 respectively. Source data are provided as a Source Data file. Center line, median; box limits, upper and lower quartiles; circles, data points; whiskers, min/max; x, mean.

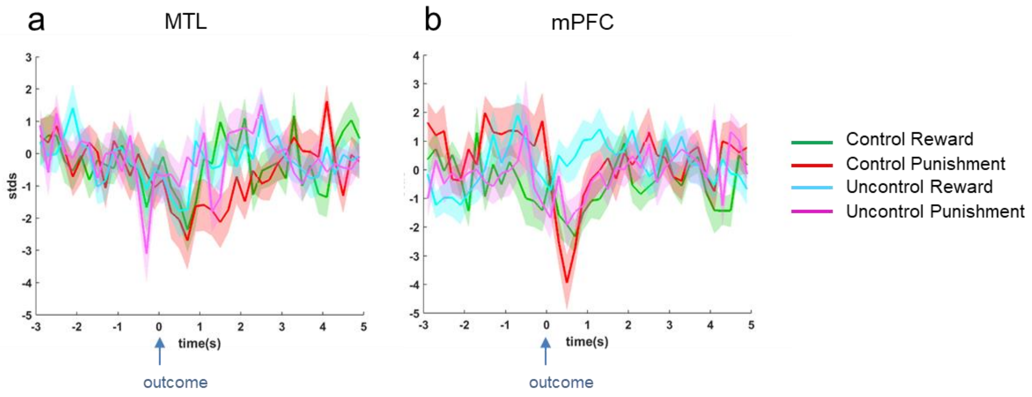

<u>Supplementary Figures 2</u>: Time course of the averaged normalized FR per outcome types. Shown for significant negatively responsive neurons for the two region-groups: MTL (a) and mPFC (b). No significant differences were found. Shaded area corresponds to SEM. N=26 MTL and 27 mPFC neurons. Source data are provided as a Source Data file. MTL = Medial Temporal Lobe, mPFC = Medial Prefrontal Cortex.

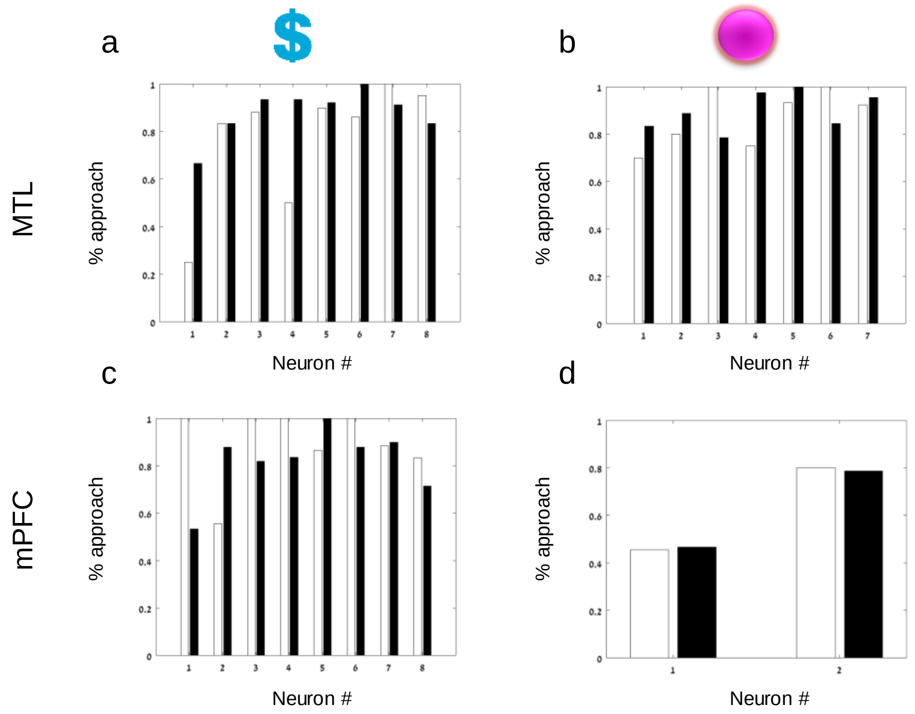

<u>Supplementary Figure 3</u>: The effect of neural responses following uncontrolled outcomes on subsequent approach behavioral choice under the HGC condition. Shown for trials where a neuron fired 200-800ms following outcome (black bars) vs. trials where a neuron did not fire (white bars). Results for **(a,b)** MTL neurons following uncontrolled reward (left) and punishment. Two-sided Mann-Whitney test, p=0.77, N = 15 neurons (7 Punishment, 8 Reward) **(c,d)** mPFC neurons following Uncontrolled Reward (left) and Punishment. N = 10 (2 Punishment ,8 Reward). MTL = Medial Temporal Lobe, mPFC = Medial Prefrontal Cortex.

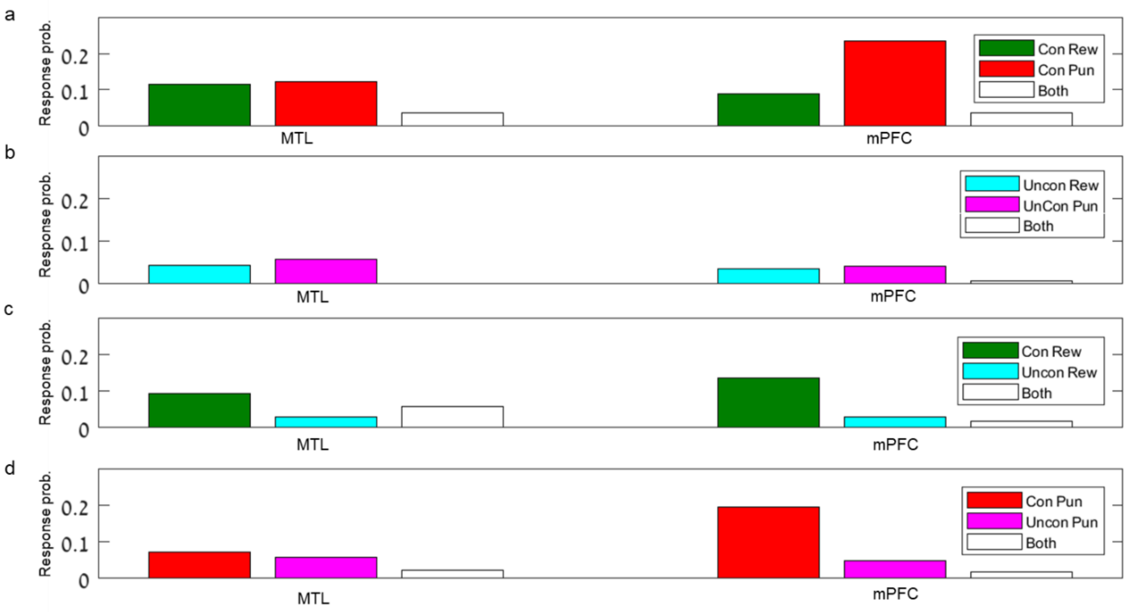

Supplementary Figures 4: Neural response probability after controlling for player's movement (# of key presses). Direct comparison between outcome conditions when the median movement of the distributions are equal **(a)** Controlled Reward Vs. Controlled Punishment, χ^2 for difference between areas, p=0.04 with N=88 neurons from two regions. **(b)** Uncontrolled Reward vs. Uncontrolled Punishment **(c)** Controlled reward vs. Uncontrolled Reward, sign rank test, p=0.864, FDR corrected, for MTL, N= 17 neurons **(d)** Controlled Punishment vs. Uncontrolled Punishment. Sign rank test, p=0.009, FDR corrected for mPFC, N= 41 neurons. MTL = Medial Temporal Lobe, mPFC = Medial Prefrontal Cortex, Con = Controlled, Uncon = Uncontrolled, Rew = Reward, Pun = Punishment.

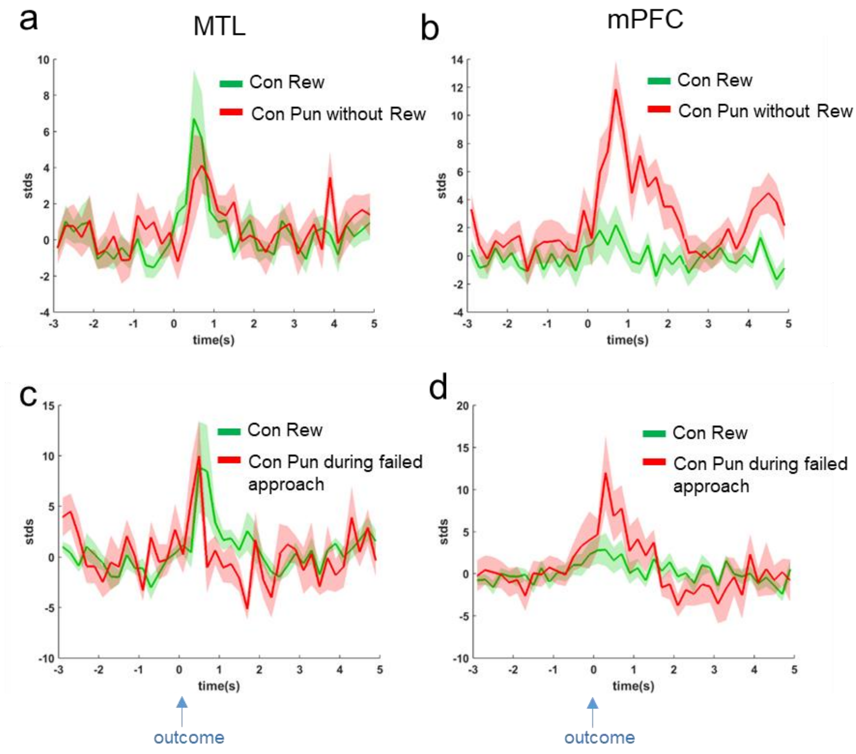

Supplementary Figure 5. **Selectivity of neural time course responses per outcome types, sorted for type of punishment outcome**. **(a, b)** neuronal responses for punishment obtained without a reward cue presence on the screen for MTL (N=15) and mPFC (N=19), respectively. **(c, d)** neuronal responses for punishment obtained following an unsuccessful approach towards a reward cue on the screen for MTL (N=8) and mPFC (N=15), respectively. Shaded area corresponds to SEM. Source data are provided as a Source Data file. MTL = Medial Temporal Lobe, mPFC = Medial Prefrontal Cortex, Con = Controlled, Rew = Reward, Pun = Punishment.

Supplementary Note 1: Neural response to cue

The number of cue trials differentiate between conditions. To account for this, we randomly selected some of the control trials to match the amount of the uncontrol trials, resulting in an equal amount of trials for all conditions. While this accounts for the statistical biases it does not account for cognitive bias as participants comprehend uncontrol conditions as more rare and thus a saliency effect may be manifested in the results.

We found 23 of 79 (29%), 27 of 61 (44%), 20 of 63 (32%) and 40 of 107 (37%) neurons that significantly responded to at least one of the four outcome conditions in the Amygdala, Hippocampus, dmPFC, CC respectively.

Examining response probability to controlled and uncontrolled cues across valence type (rewards and punishments), a higher probability to respond to the uncontrol conditions over controlled conditions was apparent in neurons from all four areas, but reach significance only for the amygdala [p=0.01 FDR corrected McNamer test]. Also, a higher probability to respond to the reward conditions over punishment conditions was apparent only for the amygdala [p=0.005 FDR corrected McNamer test]. Unlike analysis of outcome, response selectivity to valence of cue under the control condition did not differ between regions [$\chi 2=0.46$, p=0.93]. Response selectivity to valence of cue under the uncontrol condition also did not differ between regions [$\chi 2=2.1$, p=0.56].

We interperate these results in the context of saliency. As uncontrol trials are more salient compared to control trials, they evoke a stronger neural response. Similarly, as control reward trials are more salient compared to control punishment trials, they also evoke a stronger neural response. Interestingly, both these effects are strongest and only significant in the amygdala, a major hub of the brain's salience network [Seeley et al., 2007].

Supplementary note 2: Effect of Seizure Onset Zone (SOZ)

Of the 14 patients, 5 has SOZ in the MTL and 1 had an SOZ in the mPFC. This included 29 neurons in the MTL (13 amygdala, 16 hippocampus) and no neurons in the mPFC that were within the SOZ. 31 amygdala neurons were responsive to at least one of the four outcome conditions and 5 of these were within the SOZ. 26 neurons were responsive to at least on of the four conditions and 6 of these were within the SOZ. Removing these neurons from the analysis did not effect results.

Outcome response probability

While mPFC showed a clear preference to control reward over control punishment (17 vs. 4 in the dmPFC and 20 vs. 10 in the CC), MTL neurons were unbiased (10 vs. 9 in the amygdala and 5 vs. 7 in the hippocampus).

Outcome response amplitude

Following the removal of SOZ neurons (3 neurons), ANOVA analysis of neurons with increased firing in one of the four conditions found similar results to those with these neurons:

A repeated measures ANOVA with normalized FR increase following outcome (200-800msec) as the dependent variable and region-groups [MTL, mPFC], controllability (controlled/uncontrolled) and outcome-valence (reward/punishment) as the independent factors, revealed a greater response to controlled negative outcomes, specifically in the mPFC region group (3-way interaction [$F(1,53)=11.6$, $p<0.001$, $\eta^2 = 0.18$]). The ANOVA further showed that the negative bias in response to outcome was more pronounced in neurons from mPFC (2-way interaction of valence and region [$F(1,53)=4.9$, $p=0.031$, $\eta^2 = 0.09$]), and that the preferred response to controlled outcomes was more pronounced for negative valence (2-way interaction of valance and control [$F(1,53)=7.06$, $p=0.01$, $\eta^2 = 0.12$]). A main effect for control showed higher FR in response to controlled [mean=3.1, CI=(2.2,4)] compared to uncontrolled [mean=1.1, CI=(0.5,1.6)]outcomes in both region-groups [$F(1,53)=21.96$, $p<0.001$, $\eta^2 = 0.93$].

Effect of outcome response on subsequent behavior in HGC trials

after removing MTL neurons (2 responsive neurons from the left amygdala of patient 6) that were within the seizure onset zone this remained significant. We found that only MTL firing following punishment outcomes significantly correlated with behavior in subsequent HGC reward trials [beta=1.2, t=4.3, p<0.0001, FDR corrected], even after accounting for movement and time between punishment and subsequent HGC trials. Even after removing MTL neurons (2 responsive neurons from the left amygdala of patient 6) that were within the seizure onset zone this remained significant.

Comparing behavior to healthy controls

20 healthy participants (15 Females, age 32.9±3.7) performed the PRIMO task with a laptop in laboratory room as volunteers and did not receive money for performing the task. As expected, approach probability in this group was higher for the LGC condition [92.4% (±0.05)] compared to the HGC condition [78% (±0.14)] [t(19)=6.02, p<0.00001, mean difference=14%, CI=(9,19)%, Cohen's d=1.35]. Approach probabilities are relatively similar to those of our group and there was no statistical difference between this healthy population and our patients for the high [t(33)=0.94, p=0.35] and low [t(33)=0.32, p=0.75] goal conflict conditions.

healthy subjects had shorter RTs for the HGC trials (780.6±115.7) vs LGC trials (849.4± 120.6) [t(19)=3.64, p<0.002, mean=31.2, CI=(19.3,117.9), Cohen's d=0.883].
