# Supplementary material for: The role of mPFC and MTL neurons in human choice under goal-conflict
Source: Nat Commun. 2020 Jun 24;11:3192. doi: 10.1038/s41467-020-16908-z (PMC7314808; doi:10.1038/s41467-020-16908-z)
Supplement: Supplementary file 3 — Reporting Summary [file 41467_2020_16908_MOESM3_ESM.pdf]

Corresponding author(s): Talma Hendler

Last updated by author(s): May 3, 2020

Reporting Summary

Nature Research wishes to improve the reproducibility of the work that we publish. This form provides structure for consistency and transparency in reporting. For further information on Nature Research policies, see our Editorial Policies and the Editorial Policy Checklist.

Statistics

For all statistical analyses, confirm that the following items are present in the figure legend, table legend, main text, or Methods section.

n/a	Confirmed	
☐	☒	The exact sample size (*n*) for each experimental group/condition, given as a discrete number and unit of measurement
☐	☒	A statement on whether measurements were taken from distinct samples or whether the same sample was measured repeatedly
☐	☒	The statistical test(s) used AND whether they are one- or two-sided<br>*Only common tests should be described solely by name; describe more complex techniques in the Methods section.*
☒	☐	A description of all covariates tested
☐	☒	A description of any assumptions or corrections, such as tests of normality and adjustment for multiple comparisons
☐	☒	A full description of the statistical parameters including central tendency (e.g. means) or other basic estimates (e.g. regression coefficient) AND variation (e.g. standard deviation) or associated estimates of uncertainty (e.g. confidence intervals)
☐	☒	For null hypothesis testing, the test statistic (e.g. F, t, r) with confidence intervals, effect sizes, degrees of freedom and P value noted<br>*Give P values as exact values whenever suitable.*
☒	☐	For Bayesian analysis, information on the choice of priors and Markov chain Monte Carlo settings
☒	☐	For hierarchical and complex designs, identification of the appropriate level for tests and full reporting of outcomes
☐	☒	Estimates of effect sizes (e.g. Cohen's *d*, Pearson's *r*), indicating how they were calculated

Our web collection on statistics for biologists contains articles on many of the points above.

Software and code

Policy information about availability of computer code

Data collection	Intracranial EEG data was acquired using neuroport central software, in continuously updated versions up to 6.05 (BlackRock Microsystems) and behavioral data was collected through inhouse built paradigm using Java 1.6 which also ran the task.
Data analysis	Spike data was sorted using wave_clus toolbox, version 1.1 and further analysis using custom Matlav (version 2018a) scripts. Micro electrode location was identified after coregistering post-implantation CT with pre-implantation high resolution anatomical MRI using SPM12.

For manuscripts utilizing custom algorithms or software that are central to the research but not yet described in published literature, software must be made available to editors and reviewers. We strongly encourage code deposition in a community repository (e.g. GitHub). See the Nature Research guidelines for submitting code & software for further information.

Data

Policy information about availability of data

All manuscripts must include a data availability statement. This statement should provide the following information, where applicable:

- Accession codes, unique identifiers, or web links for publicly available datasets
- A list of figures that have associated raw data
- A description of any restrictions on data availability

A link to custom Matlab codes is provided:
https://github.com/tomergazit1/mPFC-and-MTL-neuronal-response-to-outcome-affects-subsequent-choice-paper-
A source data file underlying figures 4a,b,5 and supplementary figures 1,2,4,5 is provided. Other datasets are available from the corresponding author upon reasonable request.

Field-specific reporting

Please select the one below that is the best fit for your research. If you are not sure, read the appropriate sections before making your selection.

☒ Life sciences ☐ Behavioural & social sciences ☐ Ecological, evolutionary & environmental sciences

For a reference copy of the document with all sections, see nature.com/documents/nr-reporting-summary-flat.pdf

Life sciences study design

All studies must disclose on these points even when the disclosure is negative.

Sample size	single neuron activity from 14 patients with intractable epilepsy were recorded at the Tel Aviv Medical Center and at the UCLA. The final study sample included 310 units. The sample size is in the range of previously reported single unit studies. For example, Hill, Michael R., Erie D. Boorman, and Itzhak Fried. "Observational learning computations in neurons of the human anterior cingulate cortex." Nature communications 7.1 (2016): 1-12 use 358 neurons from 10 patients. Gelbard-Sagiv, Hagar, et al. "Human single neuron activity precedes emergence of conscious perception." Nature communications 9.1 (2018): 1-13 recorded 402 neurons from 9 patients. There number represent sufficient sample sizes in the community to draw conclusions in single neuron studies.
Data exclusions	The study included all patients with intracranial recordings at TASMC and UCLA who performed the PRIMO task between december 2014 and november 2019 for TASMC (11 patients) and between July to December 2016 for UCLA (4 patients). On patients from TASMC was excluded due to poor data quality
Replication	The current study report the results of an original experiment that has not been tested for replication in a new sample. Any data, code and methodological details required for replication are reported in adherence to the journal's policies.
Randomization	There was no group allocation in this study
Blinding	There was no group allocation in this study

Reporting for specific materials, systems and methods

We require information from authors about some types of materials, experimental systems and methods used in many studies. Here, indicate whether each material, system or method listed is relevant to your study. If you are not sure if a list item applies to your research, read the appropriate section before selecting a response.

Materials & experimental systems

n/a	Involved in the study	
☒	☐	Antibodies
☒	☐	Eukaryotic cell lines
☒	☐	Palaeontology and archaeology
☒	☐	Animals and other organisms
☐	☒	Human research participants
☒	☐	Clinical data
☒	☐	Dual use research of concern

Methods

n/a	Involved in the study	
☒	☐	ChIP-seq
☒	☐	Flow cytometry
☐	☒	MRI-based neuroimaging

Human research participants

Policy information about studies involving human research participants

Population characteristics	intracranial EEG was acquired from 14 patients (age = 34.8 +- 14.15, 6 females) with intractable epilepsy undergoing preoperative intracranial EEG monitoring. The study also included 20 healthy subjects (age 32.9+-3.7, 15 females).
Recruitment	All participants were recruited by their medical doctor prior to electrode implantation. Prior to participation, they were informed of the nature and purpose of this study, given a full explanation of the procedures to be followed and description of any discomfort and risk to be reasonably expected, and informed that they may decline to participate or withdraw from the study at any time without prejudice to their medical care, If they decided to participate they were required to give written informed consent by signing on the consent form approved by the institutional review board of the hospital. Participants perform the study if they are available during the intracranial monitoring, and feel well enough to participate. A self selection bias may be possible toward participants feeling better throughout the implantation or have more time (less visitors for example). This bias is not expected to effect the results as these features are less relevant to the evaluation neural response to cognitive examination.
Ethics oversight	The study conformed to the guidelines of the institutional review boards of TASMC, UCLA and Tel Aviv University

Note that full information on the approval of the study protocol must also be provided in the manuscript.

Magnetic resonance imaging

Experimental design

Design type	NA
Design specifications	NA
Behavioral performance measures	NA

Acquisition

Imaging type(s)	structural
Field strength	3 Tesla
Sequence & imaging parameters	A high resolution T1-weighted 3D (1 mm × 1 mm × 1 mm) volume was obtained using spoiled gradient echo (SPGR (MPRAGE) sequence.
Area of acquisition	whole brain

Diffusion MRI ☐ Used ☒ Not used

Preprocessing

Preprocessing software	CT to MRI co-registration using SPM12
Normalization	Non-linear normalization using SPM12 to MNI template
Normalization template	normalization was performed using SPM's tissue probability map derived from 451 T1 weighted scans, provided by the International Consortsium for Brain Mapping, John C. Mazziotta and Arthur W. Toga.
Noise and artifact removal	None
Volume censoring	None

Statistical modeling & inference

Model type and settings	None
Effect(s) tested	None

Specify type of analysis: ☒ Whole brain ☐ ROI-based ☐ Both

Statistic type for inference (See Eklund et al. 2016)	None
Correction	None

Models & analysis

n/a	Involved in the study
☒	☐ Functional and/or effective connectivity
☒	☐ Graph analysis
☒	☐ Multivariate modeling or predictive analysis
