# Supplementary material for: The role of mPFC and MTL neurons in human choice under goal-conflict
Source: Nat Commun. 2020 Jun 24;11:3192. doi: 10.1038/s41467-020-16908-z (PMC7314808; doi:10.1038/s41467-020-16908-z)
Supplement: Supplementary file 4 — Source Data [file 41467_2020_16908_MOESM4_ESM.zip › SourceFiles/Source Data FIle.pdf]

Source Data FIle

The following is a description of this data:

Figure 4a,b

We provide two excel file named: SourceFig4a_FRtablesFor4AreasUps.xlsx and SourceFig4b_FRtablesFor2AreasUps.xlsx and a Matlab file named: SourceFigure4_SuppFig2.mat. The excel sheets includes a list of positively responsive neurons and their normalized FR response for each of the 4 conditions. The Matlab file contains 2 Matlab structures:

SelectingNeuronResults4areas (relevant for Figure 4a) and SelectingNeuronResults2areas (relevant for Figure 4b)

Each of these structures contain 5 fields:

upNeuron – a list of positively responsive neurons

downNeurons – a list of negatively responsive neurons

mixedNeurons – a list of neurons with mixed polarity

upNeuronsPSTH – normalized FR per area, neuron, condition and 200ms window of positively responsive neurons. This is a cell array of size 2 or 4 (# of areas). For each area, a matrix of size: #NeuronsX4X40. Where 4 represents the 4 conditions in the following order: Control Reward, Control Punishment, Uncontrol Reward and Uncontrol Punishment and 40 represents 40 200ms windows from -3000ms to +5000ms around outcome.

downNeuronsPSTH – normalized FR per area, neuron, condition and 200ms window of negatively responsive neurons (used for Supplementary Figure 2). This is a cell array of size 2 or 4 (# of areas). For each area, a matrix of size: #NeuronsX4X40. Where 4 represents the 4 conditions in the following order: Control Reward, Control Punishment, Uncontrol Reward and Uncontrol Punishment and 40 represents 40 200ms windows from -3000ms to +5000ms around outcome.

Correction Note: Figure 4b contained a few neurons with only a black bar (fired condition). These are neurons in which the non-fired condition could not be evaluated, since a high FR caused there to be no trials where the neuron did not fire. We removed them from the figure and added an explanation to the methods, page 25. Also, some samples were mistakenly omitted

from figure 4c bottom, punishment conditions, We now added them so more red dots are apparent. These do not change the statistics or the conclusion.

Figure 5a

We provide an excel file named: SourceFig5a_timingData.xlsx detailing the normalized FR of responsive neurons in seven 200ms windows between -400 and 1000ms around the outcome for each area and condition.

Figure 5b

We provide an excel file named: SourceFig5b_EffectOnSubsequentBehaviorResults.xlsx detailing approach probability in subsequent HGC trial if neurons fired or didn't fire post outcome and their difference.

Figure 5c

We provide an excel file named: SourceFig5c_EffectOnSubsequentBehaviorResults.xlsx, detailing the differences presented in the figure.

Supplementary Figure 1

We provide an excel file named: SourceSuppFig1.xlsx detailing for each of the 15 implantation (patient was implanted twice): The number of HGC and LGC trials, number of approach trials under HGC, approach probability under HGC, response time under HGC, number of approach trials under LGC, approach probability under LGC, response time under LGC.

Supplementary Figure 2

We provide an excel file named: SourceSuppFig2_FRtablesFor2AreasDecreases.xlsx

The excel sheets includes a list of negatively responsive neurons and their normalized FR response for each of the 4 conditions. Data for this figure can also be obtained from the previously described Matlab file:

SourceFigure4_SuppFig2.mat , as described above.

Supplementary Figure 4

We provide an excel file named: SourceSuppFigure4_BalancingMovementAnalysis.xlsx

detailing the number of responsive neurons per area and condition pairs for balancing movement between conditions

Supplementary Figure 5

We provide a Matlab file: SourceSuppFigure5.mat which contain two Matlab structures:

SelectingNeuronResultsGainVSApMiss – comparing neural response to reward vs punishment during approach missed trials.

And

SelectingNeuronResultsGainVSBallsNM – comparing neural response to reward vs. punishment when rewards are not present.

Each of these structures contain 5 fields: upNeurons, downNeurons, mixedNeurons, upNeurons PSTH, and downNeuronsPSTH in a format described above in source data of figure 4a,b.
